# The relationship between hormone replacement therapy and periodontal disease in postmenopausal women: a cross-sectional study the Korea National Health and Nutrition Examination Survey from 2007 to 2012

**DOI:** 10.1186/s12903-019-0839-9

**Published:** 2019-07-15

**Authors:** Yunhee Lee, Inah Kim, Jaechul Song, Kyung-Gyun Hwang, Boyoul Choi, Seung-Sik Hwang

**Affiliations:** 1 Department of Dental Hygiene, Seoyeong University, 170, Seoyeong-ro, Wollong-myeon, Paju-si, Gyeonggi-do Republic of Korea; 2 Department of Occupational and Environment Medicine, College of Medicine, Hanyang University, 222 wangsimni-ro, Seongdong-gu, Seoul Republic of Korea; 3 Department of Dentistry/Oral and Maxillofacial Surgery, College of Medicine, Hanyang University, 222 wangsimni-ro, Seongdong-gu, Seoul Republic of Korea; 4 Department of Preventive Medicine, College of Medicine, Hanyang University, 222 wangsimni-ro, Seongdong-gu, Seoul Republic of Korea; 5 Department of Public Health Sciences, Graduate School of Public Health, Seoul National University, 1, Gwanak-ro, Gwanak-gu, Seoul Republic of Korea

**Keywords:** Hormone replacement therapy, Menopause, Periodontal disease

## Abstract

**Background:**

The purpose of this study was to investigate the relationship between hormone replacement therapy (HRT) and periodontal disease in postmenopausal women using data from the 4th and 5th Korea National Health and Nutrition Examination Survey (KNHANES).

**Methods:**

The study included data from 5,482 postmenopausal women aged 45–74 years in the 2007–2012 KNHANES. The use of female HRT for at least one month was reclassified as HRT+/HRT-. The Community Periodontal Index of Treatment Needs (CPITN) was used to assess periodontal status. Propensity score matching (PSM) was used to control selection bias, and factors affecting education, family income, and age of menopause were used as covariates in PSM. A chi-square test was used to confirm the bivariate relationship between the variables. Binary logistic regression analysis was used to adjust for confounders (age, education, family income, body mass index, age of menopause, alcohol, smoking, dental clinic visits in the past one year, use of oral care products and frequency of tooth brushing per day).

**Results:**

After adjusting for all covariates, HRT was associated with periodontal disease (OR: 0.79; 95% CI: 0.66–0.94). In particular, the relationship between HRT and periodontal disease was more evident in those with menopause under 45 years of age disease (OR: 0.55; 95% CI: 0.35–0.87).

**Conclusions:**

The results of this study supported that it is important that hormone therapy be actively considered in the policy towards postmenopausal women. Especially, health programs such as hormone replacement therapy, non-smoking, and use of oral care products are needed for women who undergo premature menopause.

## Background

The World Health Organization (WHO) defines natural menopause as 12 months of consecutive amenorrhea without an apparent cause, such as pregnancy or lactation [1]. While menopausal age varies between individuals and races, the average menopausal age for countries has been reported to be around 50 years [2]. The average menopausal age of women in Korea is 49.7 years. Considering that the life expectancy of Korean women was 85.2 years in 2015 [3], Korean women are expected to spend over one-third of their life, or approximately 35.5 years, in the menopausal state [4]. Menopause is a phenomenon that occurs naturally with aging, but postmenopausal women face increased risk of various diseases, including osteoporosis and cardiovascular diseases [5].

Postmenopausal women experience a rapid decrease in bone mineral density (BMD) due to hormone (estrogen) deficiency [6]. Meanwhile, Osteoporosis and periodontal diseases are indicative of excessive bone resorption, as both diseases have host-dependent, multifactorial causes and are regulated by local and systemic cytokines, such as IL-1 and 6, and various hormones [7]. When the periodontal status of menopausal women with osteoporosis was investigated, more severe periodontal pocket depth and attachment loss was found compared to those in the same age group who did not have osteoporosis [8]. In particular, menopause-related hormonal changes are known to affect the oral environment, due to changes in sex hormones, such as estrogen, progesterone, and testosterone, which have an impact on the secretion of proinflammatory cytokines that are involved in bone resorption [9]. Moreover, estrogen receptors in the oral mucosa react sensitively to changes in hormone levels, which leads to increased inflammation in the periodontal tissues [10].

The need for hormone replacement therapy (HRT) was presented as a means to alleviate menopausal symptoms so that a woman’s quality of life can be improved during menopause [11]. HRT plays an important role in preventing osteoporosis by reducing postmenopausal bone mass loss [12]. Menopausal women who received estrogen therapy showed a significantly increased density in their lumbar spine and femur, as compared to the control group; while, similar results were found in the alveolar bone as well [13]. Postmenopausal HRT is widely recognized to prevent osteoporosis and improve menopause-related diseases [14]. Since periodontal diseases are also affected by the state of the alveolar bone, various studies have attempted to demonstrate the association between HRT and periodontal disease [15–19]. However, the effects of HRT varied according to menopausal age and the postmenopausal period, while also showing conflicting results according to the extent of periodontal disease progression [15, 16]. In other words, HRT may have a positive effect on alveolar bone density, but it was reported to have no relationship with the attachment level of periodontal tissues and periodontal pocket depth [17–19]. Although it is believed that HRT applied to postmenopausal women was related to periodontal disease based on its mechanism, existing studies regarding the relationship between HRT and periodontal disease have produced inconsistent results. Accordingly, the objective of the present study was to identify the relationship between HRT and the risk of periodontal disease in postmenopausal women by using data from the Korea National Health and Nutrition Examination Survey (KNHANES) IV and V. The study aimed to analyze the association between HRT and the risk of periodontal diseases in menopausal women to provide basic data that can be used to establish oral health policies for menopausal women according to their life stages. For this, the study aimed to increase internal validity by using propensity score matching (PSM) to adjust for confounding factors and selection bias attributable to observational studies.

## Methods

### Study data and study population

The present study used data from the fourth (2007–2009) and fifth (2010, 2012) KNHANES conducted by the Korea Centers for Disease Control and Prevention, with exemption from review (HYI-16-195) by the Institutional Review Board (IRB) at Hanyang University. KNHANES data were collected using stratified, clustered, and systematic sampling [20]. The sampling method was adjusted for the number of households while accounting for region, type of residence, and administration district in the Republic of Korea [21]. KCDC has published the Korea Health Statistics each year, and microdata are publicly available through the KNHANES website (http://knhanes.cdc.go.kr). Based on the data from subjects who completed their health questionnaire survey and health examination, the study population was limited to menopausal women who were defined as having 12 months of consecutive amenorrhea without apparent cause, such as pregnancy or lactation [22]. Cases involving artificial menopause due to surgeries or diseases were excluded. Moreover, based on the criteria for normal menopausal age given by the Korean Society of Menopause, only women between the ages of 45 and 74 years old were included. Among the total of 5,482 women selected by the method above, a total of 2,070 women (1,035 in the HRT group and 1,035 in the non-HRT group, based on matching results) were selected for the final analysis.

### Questionnaire survey

As the dependent variable, the community periodontal index of treatment needs (CPITN) that has a score range of 0–4 points was reclassified as “No” (0–2) and “Yes” (3–4) to periodontal disease. CPITN is an index that demonstrates the need for periodontal treatment in a specific cohort or entire population of a local community, providing guidelines for understanding the status of periodontal health in a local community, establishing a periodontal treatment plan, and ensuring effective usage of periodontal treatment resources [23]. For the independent variable, the use of female HRT for at least one month was reclassified as HRT+/HRT-. The study also categorized and used the following confounding variables: general characteristics, such as age (45–54, 55–64, 65–74 years old), education (elementary and below, middle, high and above), family income (low, middle-low, middle-upper, upper), body mass index (normal, obese) and age of menopause (< 45, ≥45 years old); lifestyle habits, such as alcohol (non-drinker, past drinker, current drinker) and smoking (non-smoker, smoker); and oral care status, such dental clinic visits in the past year (yes, no), use of oral care products (yes, no), and frequency of tooth brushing per day (< 3, ≥3 times).

### Statistical analysis

**Fig. 1:**
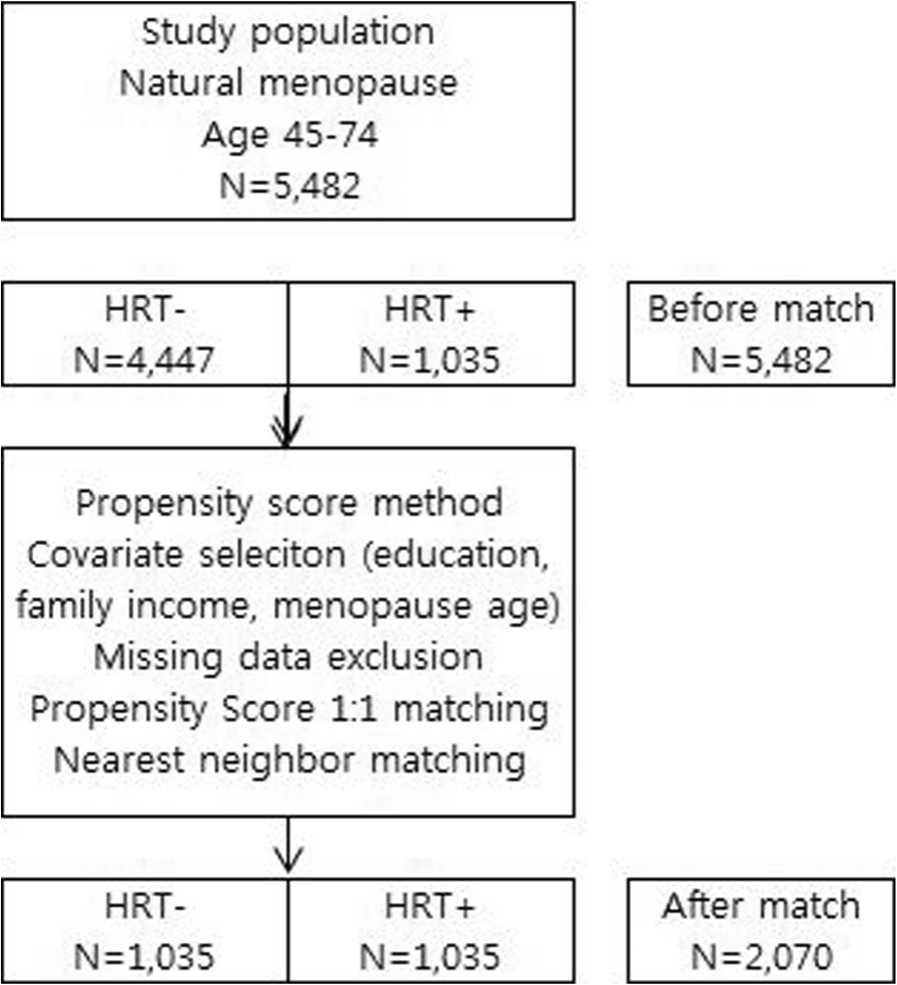
Flow chart of study population.

When analyzing the association between HRT and periodontal disease, propensity score matching (PSM) was used for balancing by controlling the covariates. Propensity score matching is a method that equates treatment and control groups on a comprehensive set of measured confounders in observational studies [24]. PSM is a single numerical value of probability that summarizes the selected confounder, using logistic regression analysis. For this analysis, HRT was the dependent variable, while education level, family income level, and age of menopause were used as covariates. Based on the propensity scores estimated in this manner, HRT+ and HRT- groups were matched 1:1 by the nearest matching neighbor. Before matching, the study population included a total of 5,482 women (4,447 in the HRT- and 1,035 in the HRT+ groups). After PSM, a total of 2,070 women were included (1,035 in the HRT+ and 1,035 in the HRT- groups) (Fig. 1).

To verify that matching was successful, differences between the confounding variables based on HRT+/HRT- were tested by the chi-square test. Moreover, binary logistic regression analysis was performed to identify the factors associated with HRT and periodontal disease, and from these results, the adjusted odds ratio (Adjusted OR) and 95% confidence interval (CI) were presented. The statistical significance level was set to *p* < 0.05. Analysis was also performed with inclusion of interaction variables to examine the interaction effects between HRT and age of menopause. STATA 11.0 (StataCorp LP, TX, USA) was used for all analyses.

## Results

### Analysis of differences before and after propensity score matching

After matching for education level, household income, and age of menopause, based on the analysis of differences before and after PSM of all study populations, pre-matching differences in covariates according to HRT+/HRT- are shown on the left side and post-matching differences are shown on the right side. Before matching, the total study population included 5,482 women; 4,447 in the HRT- group with no experience of HRT and 1,035 in the HRT+ group with previous experience with HRT. After matching, the study population was narrowed to 2,070 women, with 1,035 each in HRT- and HRT+ groups. Before matching, there was a significant difference in HRT status based on education, family income, periodontal disease, age, body mass index, drinking, dental clinic visits in the past 1 year, use of oral care products, and frequency of tooth brushing per day. After matching, there was a significant difference in HRT status based on periodontal disease, age, drinking, and use oral care products. Variables that exhibited differences based on HRT+/− prior to matching – including education level, household income, and age of menopause – no longer showed differences based on HRT+/− after matching, suggesting that PSM has been successfully completed (Table 1).

**Table 1:** Analysis of differences before and after propensity score matching

Characteristics	Group	Before matching (5,482)					After matching (2,070)				
		HRT- (4,447)		HRT+ (1,035)		×2	HRT- (1,035)		HRT+ (1,035)		×2
Education	Elementary and below	2,785	(62.63)	460	(44.44)	< 0.001***	460	(44.44)	460	(44.44)	1.000
	Middle	693	(15.58)	218	(21.06)		218	(21.06)	218	(21.06)	
	High and above	969	(21.79)	357	(34.49)		357	(34.49)	357	(34.49)	
Family income^a^	Low	1,543	(34.70)	214	(20.68)	< 0.001***	214	(20.68)	214	(20.68)	1.000
	Middle low	1,228	(27.61)	289	(27.92)		289	(27.92)	289	(27.92)	
	Middle upper	867	(19.50)	251	(24.25)		251	(24.25)	251	(24.25)	
	Upper	809	(18.19)	281	(27.15)		281	(27.15)	281	(27.15)	
Age of menopause(y)	< 45	637	(14.32)	163	(15.75)	0.242	163	(15.75)	163	(15.75)	1.000
	≥45	3,810	(85.68)	872	(84.25)		872	(84.25)	872	(84.25)	
Periodontal disease	No	2,657	(59.75)	686	(66.28)	< 0.001***	629	(60.77)	686	(66.28)	0.009**
	Yes	1,790	(40.25)	349	(33.72)		406	(39.23)	349	(33.72)	
Age (y)	45–54	990	(22.26)	265	(25.60)	< 0.001***	331	(31.98)	265	(25.60)	< 0.001***
	55–64	1,726	(38.81)	516	(49.86)		394	(38.07)	516	(49.86)	
	65–74	1,731	(38.93)	254	(24.54)		310	(29.95)	254	(24.54)	
Body mass index^b^	Normal	2,662	(59.86)	655	(63.29)	0.042*	654	(63.19)	655	(63.29)	0.964
	Obese	1,785	(40.14)	380	(36.71)		381	(36.81)	380	(36.71)	
Alcohol	No	1,459	(32.81)	266	(25.70)	< 0.001***	321	(31.01)	266	(25.70)	0.019*
	Past	1,868	(42.01)	449	(43.38)		432	(41.74)	449	(43.38)	
	Drinking	1,120	(25.19)	320	(30.92)		282	(27.25)	320	(30.92)	
Smoking	No	4,127	(92.80)	963	(93.04)	0.788	961	(92.85)	963	(93.04)	0.864
	Yes	320	(07.20)	72	(06.96)		74	(07.15)	72	(06.96)	
Dental visit < 1 years	No	3,531	(79.40)	741	(71.59)	< 0.001***	760	(73.43)	741	(71.59)	0.351
	Yes	916	(20.60)	294	(28.41)		275	(26.57)	294	(28.41)	
Use of oral care products	No	3,337	(75.04)	627	(60.58)	< 0.001***	685	(66.18)	627	(60.58)	0.008**
	Yes	1,110	(24.96)	408	(39.42)		350	(33.82)	408	(39.42)	
Frequency of tooth brushing per day	< 3	2,852	(64.13)	608	(58.74)	0.001**	581	(56.14)	608	(58.74)	0.231
	≥3	1,595	(35.87)	427	(41.26)		454	(43.86)	427	(41.26)	

*HRT* hormone replacement therapy

^a^ Income quartile. ^b^Asia-Pacific Standard: normal, less than 25 kg/m^2^; obese, more than 25 kg/m^2^

*p < 0.05, **p < 0.01, ***p < 0.001

### Comparison of existence of periodontal diseases according to the general characteristics of the subjects

The association between the general characteristics of the subjects and periodontal diseases after PSM showed statistically significant differences based on HRT status, education, family income, smoking, use of oral care products, and frequency of tooth brushing per day. The HRT- group(39.23%) showed a higher prevalence of periodontal disease than the HRT+ group(33.72%). Moreover, the prevalence of periodontal diseases was higher in those with a lower education level and household income, as well as those who were smokers, did not use oral care products, and brushed their teeth < 3 times a day (Table 2).

**Table 2:** General characteristics of the subjects and periodontal disease after propensity score matching (*n* = 2,070)

Characteristics	Group	Periodontal disease				X^2^
		No (*N* = 1,315)		Yes (*N* = 755)		
HRT status	HRT-	629	(60.77)	406	(39.23)	0.009**
	HRT+	686	(66.28)	349	(33.72)	
Age(y)	45–54	395	(66.28)	201	(33.72)	0.089
	55–64	581	(63.85)	329	(36.15)	
	65–74	339	(60.11)	225	(39.89)	
Education	Elementary and below	542	(58.91)	378	(41.09)	< 0.001***
	Middle	287	(65.83)	149	(34.17)	
	High and above	486	(68.07)	228	(31.93)	
Family income^a^	Low	255	(59.58)	173	(40.42)	< 0.001***
	Middle low	350	(60.56)	228	(39.45)	
	Middle upper	309	(61.55)	193	(38.45)	
	Upper	401	(71.35)	161	(28.65)	
Body mass index^b^	Normal	850	(64.94)	459	(35.06)	0.081
	Obese	465	(61.11)	296	(38.9)	
Age of menopause(y)	< 45	197	(60.43)	129	(39.57)	0.206
	≥45	1118	(64.11)	626	(35.89)	
Alcohol consumption	No	364	(62.01)	223	(37.99)	0.662
	Past	566	(64.24)	315	(35.75)	
	Drinking	385	(63.95)	217	(36.05)	
Smoking	No	1247	(64.81)	677	(35.19)	< 0.001***
	Yes	68	(46.58)	78	(53.42)	
Dental visit < 1 years	No	953	(63.49)	548	(36.51)	0.956
	Yes	362	(63.62)	207	(36.38)	
Use of oral care products	No	791	(60.29)	521	(39.71)	< 0.001***
	Yes	524	(69.13)	234	(30.87)	
Frequency of tooth brushing per day	< 3	726	(61.06)	463	(38.94)	0.007**
	≥3	589	(66.86)	292	(33.14)	

*HRT* hormone replacement therapy

^a^Income quartile. ^b^Asia-Pacific Standard: normal, less than 25 kg/m^2^; obese, more than 25 kg/m^2^

**p < 0.01, ***p < 0.001

### Factors associated with periodontal status

The binary logistic regression analysis regarding factors associated with the general characteristics of the subjects, periodontal disease, and HRT status after Propensity score matching showed that the HRT+ group had an adjusted OR of 0.79 (95% CI: 0.66–0.94) for those with periodontal disease, as compared to the HRT- group. The analysis including the interaction variables for interaction effects between HRT status and age of menopause after adjusting for all other factors showed that the premature menopause HRT+ group had an adjusted OR of 0.55 (95% CI: 0.35–0.87) for those with periodontal disease, as compared to the premature menopause HRT- group, while the adjusted OR for those with periodontal disease was 0.71 (95% CI: 0.50–1.00) and 0.60 (95% CI: 0.42–0.85) in the normal menopause HRT- and HRT+ groups, respectively. The upper household income group had an adjusted OR of 0.74 (95% CI: 0.55–0.99) for those with periodontal disease, as compared to the low household income group. Meanwhile, the adjusted OR for those with periodontal disease in those who responded “Yes” to smoking and the use of oral care products was 2.04 (95% CI: 1.44–2.89) and 0.78 (95% CI: 0.63–0.95), respectively (Table 3).

**Table 3:** Association between HRT and periodontal disease (*n* = 2,070)

Variable		Adjusted OR	(95% CI)
HRT	HRT-	1.000	
	HRT+	0.79**	0.66–0.94
Age_menopause #HRT_status	< 45#HRT-	1.000	
	< 45#HRT+	0.55*	0.35–0.87
	≥45#HRT-	0.71*	0.50–1.00
	≥45#HRT+	0.60**	0.42–0.85
	45–54	1.000	
Age	55–64	1.09	0.86–1.38
	65–74	1.08	0.82–1.42
Education	Elementary and below	1.000	
	Middle	0.85	0.66–1.09
	High and above	0.85	0.67–1.08
Family income	Low	1.000	
	Middle low	1.05	0.81–1.37
	Middle upper	1.08	0.82–1.44
	Upper	0.74*	0.55–0.99
Bmi	Normal	1.000	
	Over	1.09	0.90–1.32
Alchol	No	1.000	
	Past	0.93	0.75–1.16
	Dringking	0.93	0.73–1.20
Smoking	No	1.000	
	Yes	2.04***	1.44–2.89
Dental visit < 1 years	No	1.000	
	Yes	1.12	0.91–1.38
Use of secondary oral products	No	1.000	
	Yes	0.78*	0.63–0.95
Frequency of tooth brushing per day	< 3	1.000	
	≥3	0.85	0.70–1.02

*HRT* hormone replacement therapy

^a^ Income quartile ^b^Asia-Pacific Standard: normal, less than 25 kg/m^2^; obese, more than 25 kg/m^2^

*p < 0.05, **p < 0.01, ***p < 0.001

## Discussion

After adjusting for various potential factors associated with periodontal diseases, the study found that HRT and periodontal diseases were associated. The analysis of menopausal women aged between 45 and 74 years old showed that the HRT+ group was less likely to develop periodontal diseases than the HRT- group, which supported the results of previous studies. In a case-control study by Haas et al. that examined Brazilian women aged 40~69 years old, the likelihood of periodontal diseases in the group who did not undergo HRT during menopause was 2.10 times higher than the pre-menopausal women; while, the likelihood of the group who underwent HRT during menopause was 1.11 times higher than the pre-menopausal women. These results show a similar context to the present study and HRT may be viewed as an option for lowering the risk of exacerbating periodontal status. However, a study by Pizzo et al. evaluating 91 Italian menopausal women aged 50~62 years reported that there was no difference in periodontal pocket depth between the group who underwent HRT and the group who did not, but the group who did not undergo HRT had a higher plaque level. A case-control study by López-Marcos et al. [25] on 210 Spanish menopausal women aged 40–58 years reported that the estrogen patch group showed a reduction in periodontal pocket depth, but since an association with receding gums could not be found, further consideration was needed on this topic. These conflicting study results may exist because of varying clinical assessment tools for age group, menopausal period, and periodontal status.

According to precedent studies, subjects in the same age group showed differences in the effects of HRT depending on the menopausal period. Singh et al. [26], Richa et al. [27] reported that the effects of estrogen were greater in people with a longer menopausal period and lower bone mass. Meanwhile, women with premature menopause occurring before the average menopausal age of 50 were reported to have a higher risk of osteoporosis, cardiovascular disease, and associated mortality rate than women with normal menopause due to having a longer period of estrogen deficiency [28]. A prospective study by Women’s Health Initiative (WHI) compared BMD after dividing the subjects into groups of menopausal age of < 40 and ≥ 40 years. The results showed that the < 40-year-old group had a statistically significantly lower BMD and the risk of fracture was also higher in the premature menopause group [29]. Sullivan et al. [30] reported that among women who did not undergo HRT, those who experienced menopause at an earlier age tended to have a high risk of fracture and reduction in BMD, while Faubion et al. [31] reported that women with early estrogen deficiency had the need for estrogen therapy with a higher dose due to increased risk of reduction in BMD and death than normal menopausal women.

In the present study, the effects of HRT were more evident in women with premature menopause, before the age of 45 years, which supported the findings in existing studies. This occurs because the effects of HRT are more prominent in premature menopausal women because they have a lower BMD for a longer period of estrogen deficiency, which was consistent with a 3-year prospective study by PEPI, in which the patient age and pre-treatment BMD were the factors associated with an increase in post-HRT BMD [32].

In the present study, In this study, the ‘upper’ household income group exhibited lower prevalence of periodontal disease, while smokers and users of oral hygiene products exhibited higher prevalence of periodontal disease. Such results were similar to the findings in studies by Schuch et al. [33] where people with a higher education level and income tended to have a higher rate of undergoing treatment for periodontal disease.

MacFarlane et al. [34] have reported that patients with chronic periodontal disease showed impaired phagocytic function compared to those who managed the average adhesion an ingestion of polymorphonuclear leukocytes. Reduction in BMD caused by smoking is especially evident in menopausal women [35]. These results are connected to the effects of HRT appearing more prominently when the periodontal status is poorer. Based on the aforementioned results, it is determined that for premature menopause, HRT should be seriously considered due to an increased risk of bone loss and periodontal disease caused by prolonged estrogen deficiency. Moreover, HRT may be more effective in premature menopausal women with a poor periodontal status.

The present study aimed to be highly representative of Korea by using the KNHANES data that measured the periodontal status according to WHO criteria. However, because it was a cross-sectional study, there were limitations because causal relationships between the influencing factors and the onset of periodontal disease could not be identified and the duration of HRT was not considered. Compared to existing studies, the significance of the present study can be found in that it used CPITN, an objective periodontal status assessment tool, and that various factors such as age, age at menopause, and oral care behavior were adjusted to analyze the association between HRT and periodontal disease with consideration for a more diverse set of factors.

## Conclusions

The present study was performed to identify the association between HRT and periodontal disease in menopausal women in Korea by using the data from the KNHANES IV and V. The study used CPITN as an objective assessment tool for periodontal status and adjusted for various factors, such as age, menopausal age, and oral care behavior to more definitively identify the association between HRT and periodontal disease. The results showed that HRT was associated with a lower risk of periodontal disease. Such effect was more evident in women who experienced premature menopause, did not maintain proper oral care, had a lower income level, and were smokers. Therefore, when establishing oral health policies for menopausal women, serious consideration should be given to HRT. Moreover, healthcare programs involving HRT, regular dental check-ups, and oral care need to be implemented especially for premature menopausal women who are smokers.

## Data Availability

The dataset used and/or analyzed during the current study available from the corresponding author on reasonable request.

## Abbreviations

CPITN: Community periodontal index of treatment needs
HRT: Hormone replacement therapy
KNHANES: Korea national health and nutrition examination survey
PSM: Propensity score matching
